# EEG correlates of sensorimotor processing: independent components involved in sensory and motor processing

**DOI:** 10.1038/s41598-017-04757-8

**Published:** 2017-06-30

**Authors:** Andrew Melnik, W. David Hairston, Daniel P. Ferris, Peter König

**Affiliations:** 1 Institute of Cognitive Science, University of Osnabrück, Osnabrück, Germany; 2 Human Research and Engineering Directorate, U.S. Army Research Laboratory, Adelphi, MD USA; 3 School of Kinesiology, University of Michigan, Ann Arbor, MI USA; 4 Department of Neurophysiology and Pathophysiology, University Medical Center Hamburg-Eppendorf, Hamburg, Germany

## Abstract

Sensorimotor processing is a critical function of the human brain with multiple cortical areas specialised for sensory recognition or motor execution. Although there has been considerable research into sensorimotor control in humans, the steps between sensory recognition and motor execution are not fully understood. To provide insight into brain areas responsible for sensorimotor computation, we used complex categorization-response tasks (variations of a Stroop task requiring recognition, decision-making, and motor responses) to test the hypothesis that some functional modules are participating in both sensory as well as motor processing. We operationalize functional modules as independent components (ICs) yielded by an independent component analysis (ICA) of EEG data and measured event-related responses by means of inter-trial coherence (ITC). Our results consistently found ICs with event-related ITC responses related to both sensory stimulation and motor response onsets (on average 5.8 ICs per session). These findings reveal EEG correlates of tightly coupled sensorimotor processing in the human brain, and support frameworks like embodied cognition, common coding, and sensorimotor contingency that do not sequentially separate sensory and motor brain processes.

## Introduction

In everyday life, humans respond to a variety of stimuli to produce a range of motor commands in diverse contexts. Object manipulation, object avoidance, social interaction, and navigation are all examples of daily sensorimotor processing. Complex speeded-response tasks, which often take about 500 ms in response latency^1^, provide an opportunity to trace activation of brain areas involved in sensorimotor processing using brain imaging techniques such as electroencephalography (EEG) or magnetoencephalography (MEG). These two brain imaging techniques, in particular, are helpful for research on human sensorimotor processing as they are noninvasive yet provide fine temporal resolution. Past studies have identified cortical areas characterised as being specialised for sensory recognition or motor execution^2^, but we know less about the brain processes involved in moving from sensor recognition to motor execution. From a theoretical perspective of sensorimotor control, it would be very beneficial to identify areas (or modules) specifically involved in turning sensory information into motor actions during sensorimotor processing.

A classical view conceptualises sensorimotor processing as a series of functional modules activated in a sequence from sensory input to motor execution. For a usual lab setup with stimulus presentation and response, such a view implies that all functional modules participate in sensorimotor processing only once. Presumably, functional modules related to sensory processing do not have a direct influence on action execution. Such a classical view also implies that action-related modules do not influence perception. This classical view explains many experiments on monkeys with straightforward stimulus-response paradigms in perceptual discrimination and value-based decision tasks^3, 4^, and provides a framework that “describes how to form decisions using priors, evidence, and value to achieve certain desirable goals”^5^.

Other views of human brain function propose that sensory and motor processing are intimately related^6, 7^. Embodied cognition^8^ supports the ideas that perception guides motor actions, motor actions influence perception, and brain concepts are fundamentally grounded in both perception and action^9^. This framework is supported by experimental findings of action intentions influencing visual processing^10^, motor-visual attentional priming^11^, and visuo-motor priming^12^. The common-coding approach implies that “there are certain products of perception on the one hand and certain antecedents of action on the other that share a common representational domain”^13^. This view proposes similarity between afferent and efferent codes instead of a separate-coding-and-translation approach^14^. Some have claimed that the approach is more powerful than the traditional approach to the perception and action relationship, which invokes separate coding rather than common coding^13^ in explaining effects like negative asynchrony^15, 16^ and stimulus-response compatibility^17, 18^. Lastly, sensorimotor contingency theory emphasises the key role of action for perception. “Recognition of an object occurs, not when neural excitation due to the object arrives in some cortical area, but when we are exercising our mastery of the way the object behaves under exploratory manipulation”^19^. Sensorimotor contingency serves as the theoretical foundation of sensory substitution, when visual sensation is perceived through touch^20^ or learning a new sense with a sensory augmentation device^21–23^. All three of these different frameworks support the idea that sensorimotor processing can be a holistic process rather than a series of separate modules.

In the present study, we examined the neural substrates of sensorimotor processing in humans using a complex cognitive task and speeded-categorization response. We investigated whether we could consistently find functional modules participating in both sensory processing and motor processing. We operationalized functional modules as independent components (ICs) yielded by an independent component analysis (ICA) of EEG data, resulting in decomposition of many electrode channels recorded in parallel into presumed independent sources of brain activity^24–26^. The classical view in its pure form leads to the hypothesis that activity of functional modules is strictly related to either sensory stimulation or motor response, but not to both. The alternative frameworks propose that some functional modules will relate to both sensory stimulation and motor response. These modules would likely participate for the whole sensorimotor process, from sensory recognition to motor response. In this study, we tested these competing hypotheses to provide insight into the theoretical constructs of sequential processing and the alternative frameworks (embodied cognition, common coding, and sensorimotor contingency).

The current study revealed demonstrable EEG correlates of sensorimotor processing in the human brain. Our experimental paradigm used stimulus presentation and speeded categorization-response tasks which required recognition, decision, and motor response. A stimulus consisted of three sources of information about colour which appeared simultaneously: the written name of a colour, the hue of the font, and the pronounced word. The tasks were either to detect a specific colour or to compare different sources of information about colour and then to respond by pressing the appropriate key. We measured event-related responses by means of inter-trial coherence (ITC)^27, 28^. We consistently found independent components with event-related ITC responses related to both sensory stimulation and motor response onsets. We refer to such independent components as Sensorimotor ICs. In order to check whether the number of channels in an EEG system influences a number of Sensorimotor ICs, we recorded subjects with both 64- and 127- channel EEG systems, and additionally analysed a subset of 32 channels (from the 64-channel EEG recordings). We found that the number of channels does not influence the number of Sensorimotor ICs. We conclude that EEG data support frameworks of sensorimotor processing that directly couple sensory recognition and motor responses rather than sequentially separate them.

## Results

### Introduction to the design of the experiment

We recorded healthy students in the EEG experiment while they were performing speeded categorization-response tasks (variations of a Stroop task). Subjects completed motor responses by pressing one of two buttons on a keyboard. EEG data were filtered with a 3–45 Hz bandpass finite impulse response filter. After elimination of noisy periods, we processed EEG data by ICA using EEGLAB’s ‘runica’ function^24–26^. Further data analysis and results were based on the resulting ICs and their activity. In order to measure sensory and motor related activity of ICs, we calculated the ITC of all trials in a recording session twice per independent component. The first ITC calculation was done with stimulus alignment of trials, and the second ITC calculation was done with button-press alignment of trials. The maximum value of ITC in a sensory time-frequency window of interest was assigned as the Sensory ITC value of the IC (see Methods for details). The maximum value of ITC in a motor time-frequency window of interest was assigned as the Motor ITC value of the IC. Thus, each IC had sensory-related and motor-related ITC values, which served as scalar measures of sensory and motor event-related responses of an IC, respectively. We used these values as indicators of EEG correlates of sensorimotor processing in the human brain. In order to investigate localization of ICs, an equivalent dipole (ED) was calculated for each IC. See the Methods section for more details about subjects, stimuli, tasks, data acquisition, and data processing.

### Artefact-related ICs rejected from data analysis

**Figure 1:**
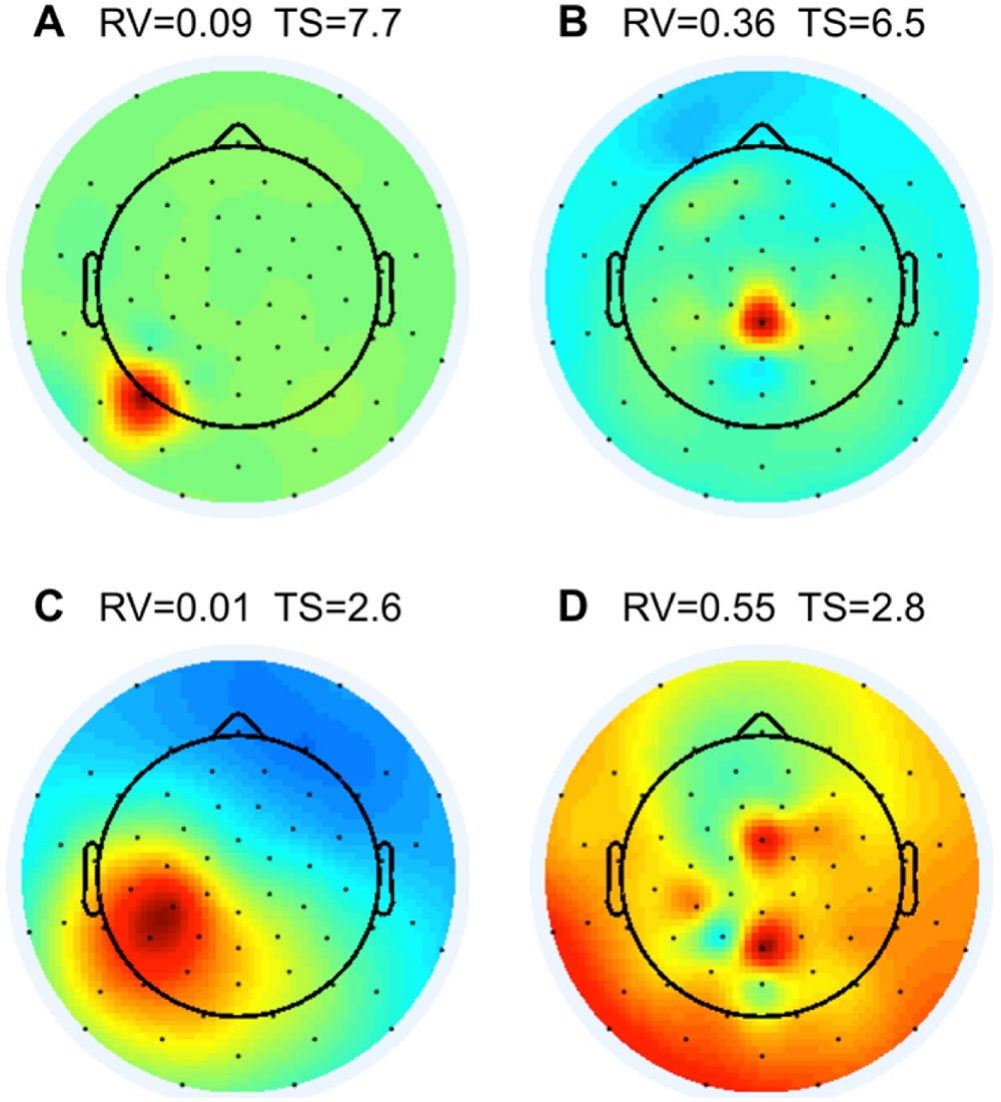
Examples of (**A**,**B** and **D**) artefact ICs which were excluded and (**C**) a good IC kept for further analysis. The black dots are positions of channels in the 64-channel EEG system. Colour-coding indicates projection weights of an IC (column of the inverse weight matrix, EEG.icawinv in EEGLAB). Panels A and B demonstrate ICs with topographical sparseness (TS) > 5, that were excluded from further analysis. Panels B and D demonstrate ICs with residual variance (RV) of the equivalent dipoles exceeding 15%. These ICs were also excluded from further analysis. Panel C demonstrates an IC which was kept for further analysis (TS > 5 and RV < 15%).

**Figure 2:**
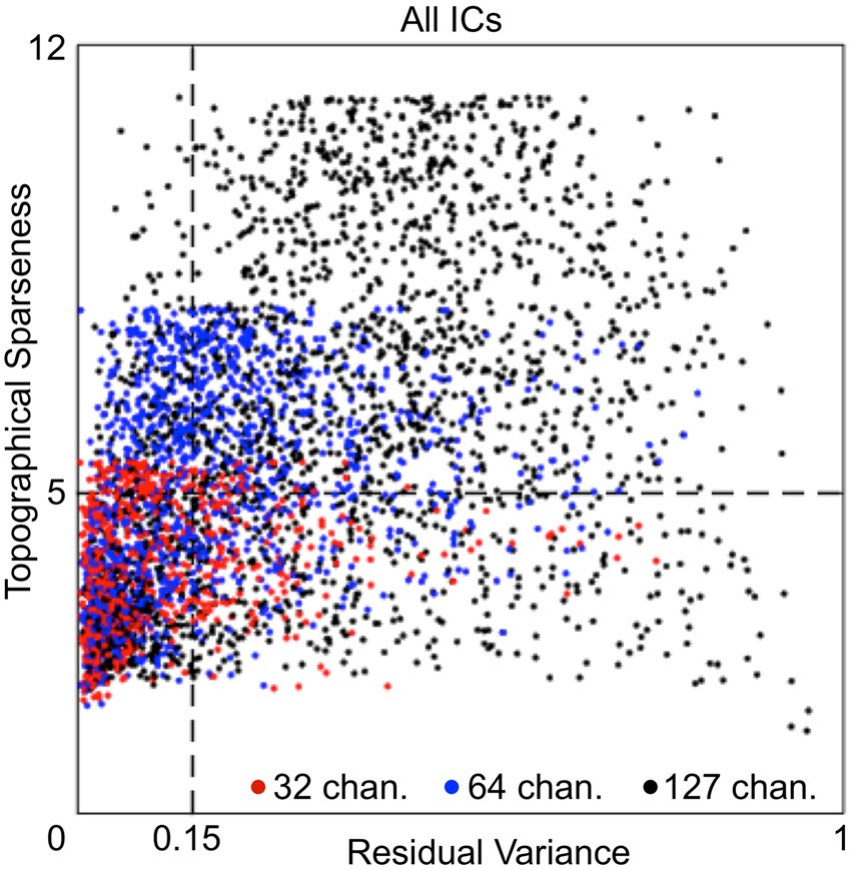
Scatter plot of Topographical Sparseness and Residual Variance values of all ICs in the study. Each dot represents an IC. X-axis = residual variance of an equivalent dipole of the IC. Vertical dotted line shows the residual-variance threshold = 0.15 (15%). Y-axis = topographical sparseness (TS) of an IC. Horizontal dotted line shows the TS threshold = 5. The number of all ICs from all recorded sessions in the study amounts to 4169 ICs, which consist of the following three groups: 672 ICs from 32-channel datasets (red dots), 1344 ICs from 64-channel datasets (blue dots), and 2153 ICs from 127-channel datasets (black dots). ICs in the lower left rectangle in the scatter plot were selected for further data analysis.

We identified artefact ICs, which were presumably not of neuronal origin, using two measures. The first measure of artefact-related ICs was the residual variance (RV) of the equivalent dipole. If the residual variance of the equivalent dipole exceeded 15%, then the corresponding IC was marked as residual-variance-bad^29–31^ and excluded from data analysis. Figure 1B and D show such examples of excluded ICs. Figure 2 also shows ICs with residual variance greater than 15% on the right of the vertical dotted line. In contrast, Fig. 1C shows an examples of IC with residual variance less than 15% that was valid for further data analysis.

The second measure of artefact-related ICs was topographical sparseness (TS). When a topography of an IC is widely distributed (Fig. 1C), then topographical sparseness is low. When a topography of an IC is concentrated over a small area (e.g., over one electrode, as shown in Fig. 1A), then topographical sparseness is high. We assume that ICs with high topographical sparseness do not represent a source in the brain, but may arise due to a change in contact properties between an electrode and skin during the experiment, bad channels, or other electrode related artefacts. Our topographical sparseness measure was calculated for each IC using the following formula: TS = max(abs(PW-mean(PW)))/std(PW), where PW is a vector of projection weights (PW) of an IC (column of the inverse weight matrix, EEG.icawinv). We obtained the EEG.icawinv matrix using EEGLAB’s ‘runica’ function^24–26^. The max(x) function returns the largest element in x. The abs(x) function returns absolute values of elements in x. The mean(x) function returns the mean value of elements in x. The std(x) function returns the standard deviation of elements in x.

This value of topographical sparseness captures the sparseness of projection weights of an IC and is dependent on the number of EEG channels. For example, TS = 5.5 for a vector of 32 EEG channels with only one non-zero element (e.g., [1,0,0,…]) but TS = 7.9 for a vector of 64 EEG channels with only one non-zero element and TS = 11.2 for a vector of 127 EEG channels with only one non-zero element. Achieving a constant TS = 5.5, 32 EEG channels would need one non-zero element, 64 EEG channels would need two non-zero elements, and 127 EEG channels would need four non-zero elements. The size of an area covered by one electrode in an EEG system with 32 channels is approximately equal to a size of an area covered by two electrodes in an EEG system with 64 channels, and 4 electrodes in an EEG system with 127 electrodes.

ICs with the topographical sparseness value lower than 5 have a size of a contributing area exceeding one-electrode coverage in 32 channels EEG system. Therefore, we excluded from data analysis all ICs with the topographical sparseness value higher than 5. Figure 1A and B show such examples of excluded ICs. Figure 2 also shows excluded ICs over the horizontal dotted line that had topographical sparseness values higher than 5.

**Figure 3:**
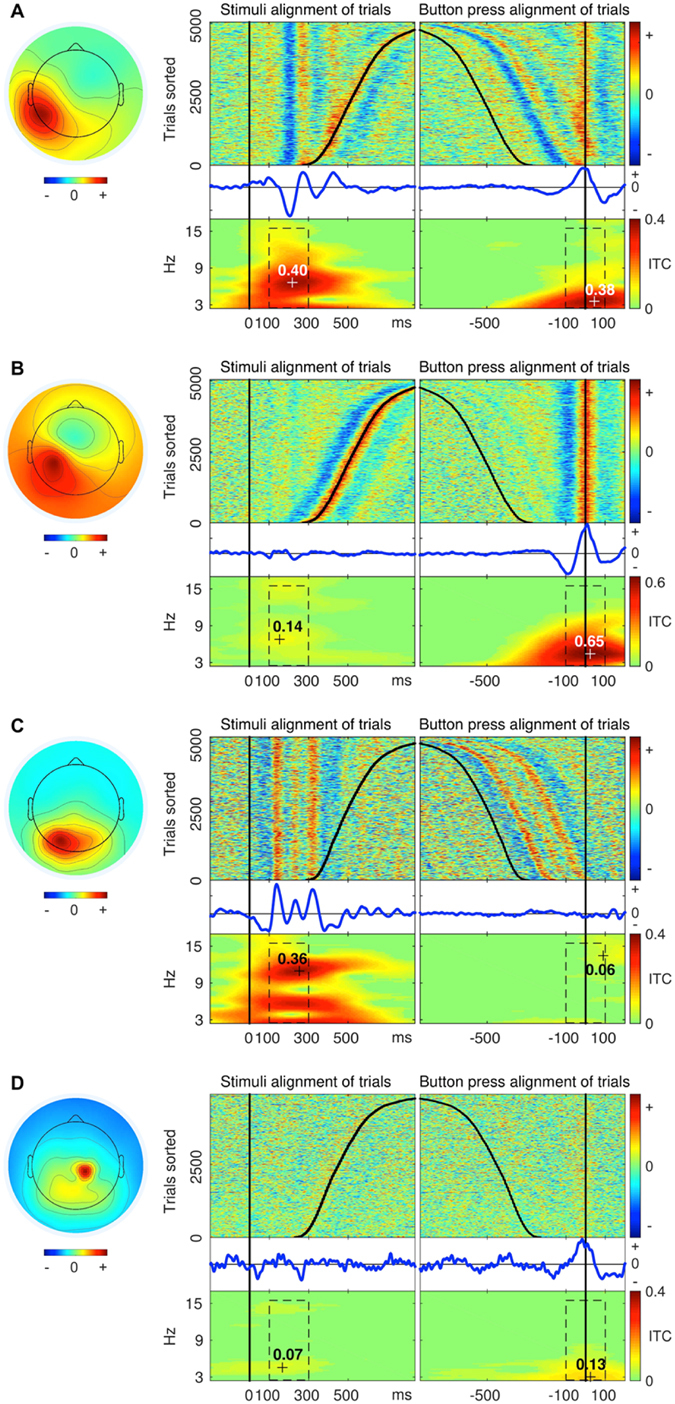
Examples of (**A**) Sensorimotor, (**B**) Motor, (**C**) Sensory, and (**D**) Unspecified ICs. The exact procedure of grouping of all ICs into the four groups is described below. The round component scalp maps on the left side represent topography of ICs from 64-channel EEG recordings. The component scalp map values returned by ICA were proportional to µV (scaling is distributed between the component maps and activity time courses)^26, 33^. Panels on the right side of each scalp map depict activity data of the IC. (1) The “top-left” plot in each panel shows colour-coded amplitude of the IC activity in a recording session. Each line in the plot represents a trial. Trials were sorted according to latency of reaction time. The black vertical line (OX = 0 ms) shows the onsets of the stimuli and the black curve in the positive direction shows the moments of a button-press event. (2) The “top-right” plot in each panel depicts the same trials as the “top-left” plot, but this time trials were aligned by onset of a button-press event (OX = 0 ms). The straight vertical line shows the moments of the button-press events and the curve in the negative direction shows the onsets of the stimuli. (3) The “middle-left” and (4) “middle-right” plots in each panel show Event Related Potentials (blue curves) derived from trials depicted in the plots above (“top-left” and “top-right”). (5) The “bottom-left” plot in each panel shows inter-trial coherence (ITC) of trials from the “top-left” plot. The black dashed rectangle represents the time-frequency window of 100 ms to 300 ms by 3 Hz to 15 Hz. The maximum value of ITC in this time-frequency window represents Sensory ITC value of the IC. (6) The “bottom-right” plots in each panel show ITC of trials from the “top-right” plot. The black dashed rectangle represents the time-frequency window of −100 ms to 100 ms by 3 Hz to 15 Hz. The maximum value of ITC in this time-frequency window represents a Motor ITC value of the IC.

We obtained a total of 1412 accepted ICs (the lower left rectangle in the scatter plot in Fig. 2) and 2757 rejected ICs. The ICA method automatically produces the same number of ICs as the number of channels in EEG data (e.g., 127 ICs from 127-channel data). A systematic analysis of ICA with different numbers of EEG electrodes found that robust “electrocortical sources can be well captured using an electrode montage with as few as 35 channels”^32^. Therefore many ICs in a 127-channel collection may contain information not relevant for the experimental paradigm. From 32-channel datasets (red dots in Fig. 3), 419 ICs were accepted and 253 ICs were rejected. This was, on average, 19.95 ICs accepted per session and 12.05 ICs rejected per session. From 64-channel datasets (blue dots in Fig. 3), 529 ICs were accepted and 815 ICs were rejected. This was, on average, 25.19 ICs accepted per session and 38.81 ICs rejected per session. From 127-channel datasets (black dots in Fig. 3), 464 ICs were accepted and 1689 ICs were rejected. This was, on average, 27.29 ICs accepted per session and 99.35 ICs rejected per session. More than half of the rejected ICs are from 127-channel datasets and less than 10% are from 32-channel datasets. This suggests that after a certain number of channels, increasing the number of channels does not result in the detection of additional independent sources of brain activity in stationary subject conditions using the experimental paradigm in this study. More unconstrained human behaviours in varying environmental conditions with substantive head motion would likely benefit from a higher number of EEG channels^32^.

### Measuring sensory and motor event-related responses of ICs

To address the question of whether activity of some ICs relates to both sensory stimulation and motor response, we calculated event-related responses two times for each IC in each recording session (Fig. 3). The first time was for sensory stimulus alignment of trials and the second time was for motor response alignment of trials (button press). Varying reaction time among trials in a session allowed us to differentiate stimulus and button-press event-related responses by extracting epochs either by stimulus onsets or by motor response onsets (button-press events).

In order to estimate to what extent each IC represents sensory and/or motor processing, we calculated ITC of trials epoched separately by sensory and motor onsets. Figure 3A shows an example of an IC with clear event-related responses for both sensory and motor alignment of trials. Aligning the approximately 5000 trials of a recording session to the stimulus onset revealed a sensory event-related ITC response. The maximal value in the time-frequency window of interest of ITC (see details in Methods) served as a scalar measure of the sensory event-related response of the IC. In the example in Fig. 3A, this Sensory ITC value was equal to 0.4. Likewise, aligning the same trials on motor onset revealed a motor event-related ITC response. The maximal value in the time-frequency window of interest of ITC served as a scalar measure of the motor event-related response of the IC. In the example in Fig. 3A, the Motor ITC value was equal to 0.38. Figure 3B shows an example of an IC with motor event-related response only. Figure 3C shows an example of an IC with sensory event-related response only. Figure 3D shows an example of an IC with neither sensory nor motor event-related responses. Thus, each IC in the study had two ITC values (sensory and motor) which identified the extent that the IC represented sensory or motor processing.

### Four groups of ICs: Motor, Sensory, Sensorimotor, and Unspecified

**Figure 4:**
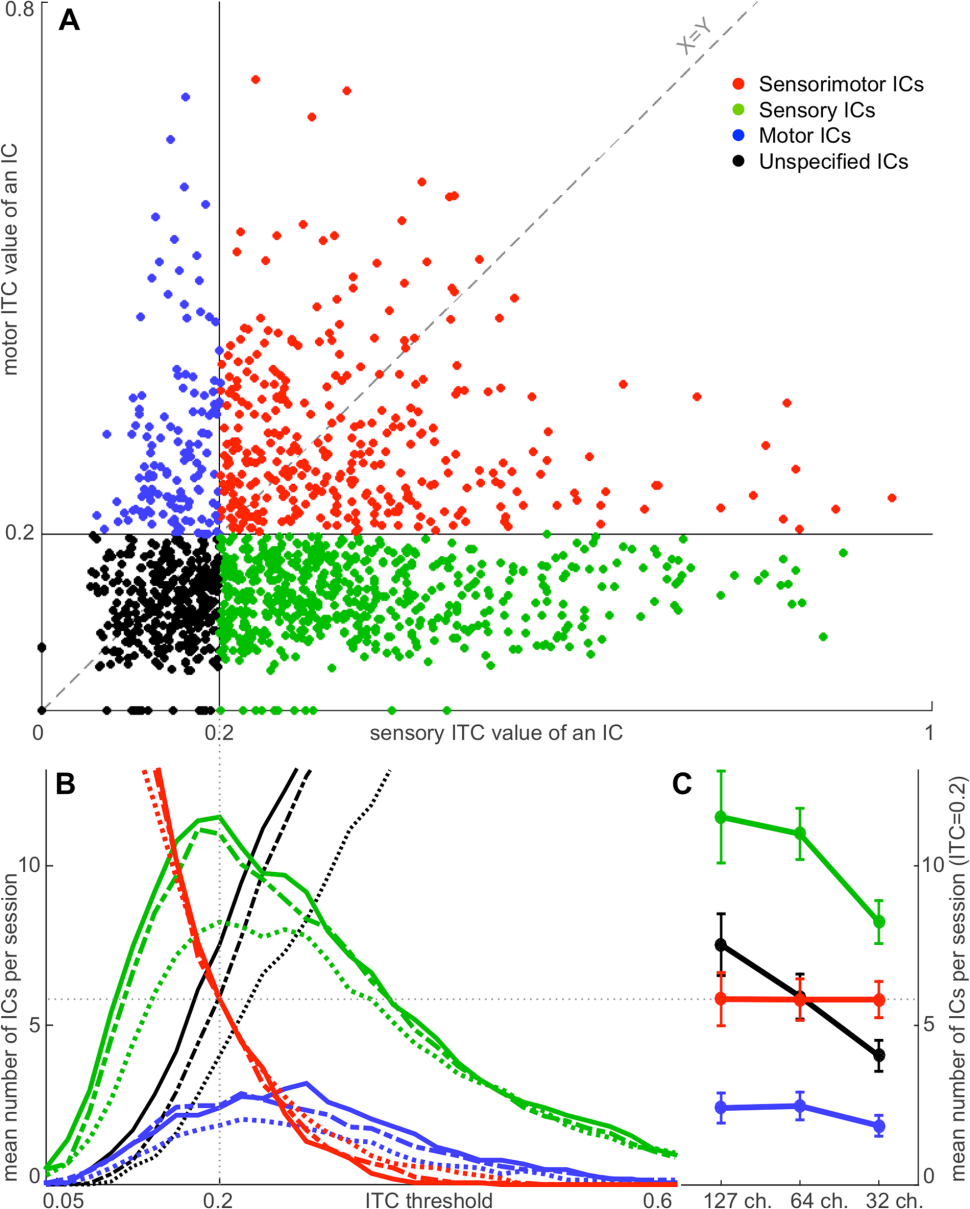
Distribution of ICs into the four groups: Motor, Sensory, Sensorimotor, and Unspecified. Panel (A) depicts 1412 accepted ICs in the study from 32-, 64- and 127-channel datasets. Each dot represents an IC with a Sensory ITC value as the X coordinate and a Motor ITC value as the Y coordinate. Blue dots represent Motor ICs. Green dots represent Sensory ICs. Red dots represent Sensorimotor ICs. Black dots represent Unspecified ICs. The vertical black line at X = 0.2 represents the sensory ITC threshold, and the horizontal black line at Y = 0.2 represents the motor ITC threshold to separate ICs into the four groups. The grey diagonal dashed line (X = Y) represents the ITC-threshold trajectory of the intersection of the threshold lines. The results of distribution of ICs into the four groups at different points of the trajectory are shown in Panel (B). The X-axis is derived from the X = Y dashed line in Panel (A). The Y-axis represents a mean number of ICs in a group per session at different points of the grey diagonal dashed ITC-threshold-trajectory line (X = Y) in Panel (A). The grey dotted line at X = 0.2 indicates the selected ITC threshold for Sensory and Motor ITC values in Panel (A). X-axis data points interval in the illustration is equal to 0.02. Red curves represent Sensorimotor ICs. Green curves represent Sensory ICs. Blue curves represent Motor ICs. Black curves represent Unspecified ICs. Dotted curves represent 32-channel datasets. Dashed curves represent 64-channel datasets. Solid curves represent 127-channel datasets. Panel (C) shows the projection of the intersection points of the 12 coloured lines in Panel (B) with the grey dotted line at X = 0.2. Y-axis and colour coding remain the same as in Panel (B). Error bars represent standard error of the mean.

**Figure 5:**
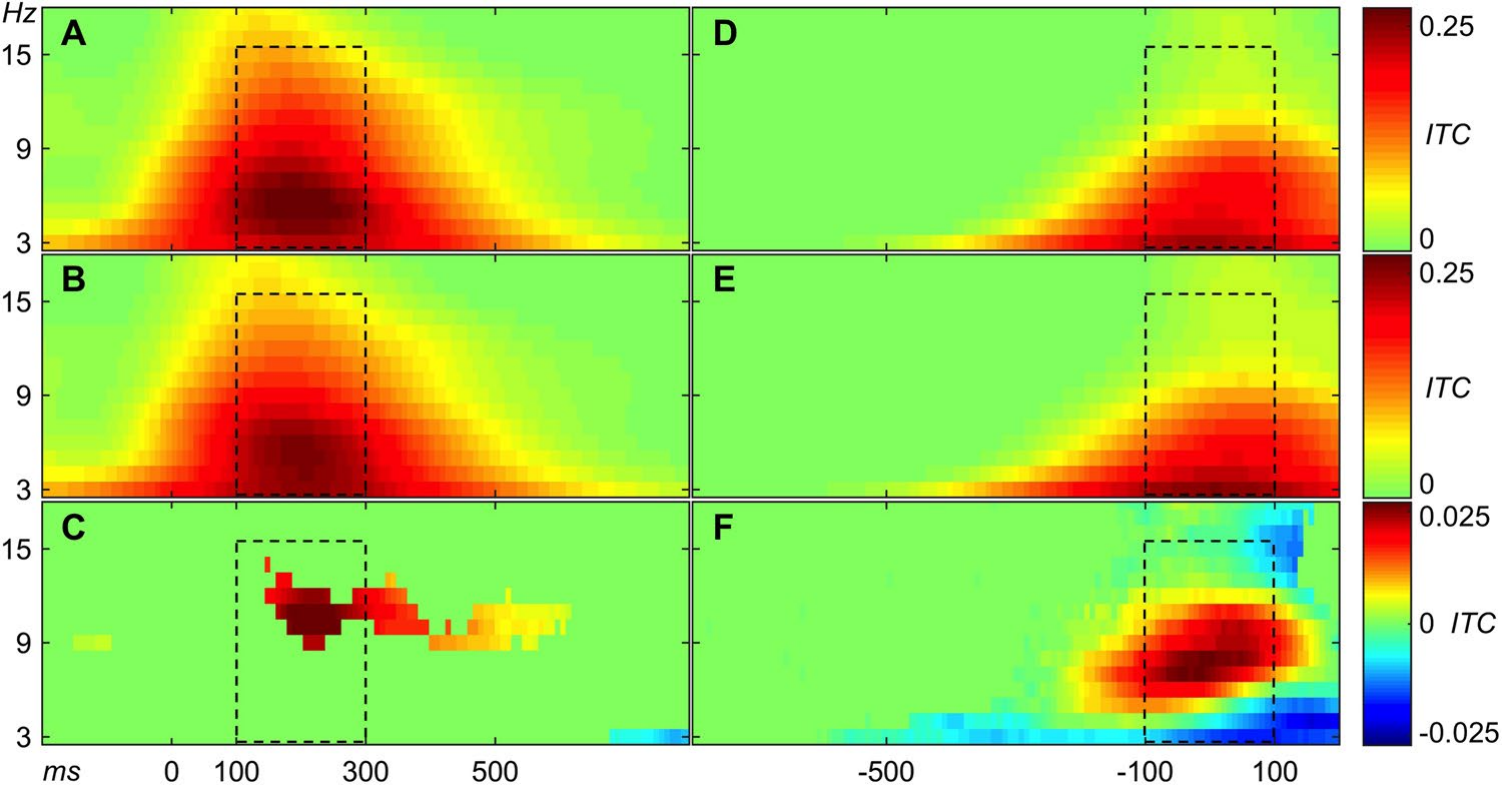
(**A**) Average ITC over 600 Sensory ICs. (**B**) Average ITC over 343 SensoriAverage ITC plots of Sensory, Motor, and Sensorimotor ICs. motor ICs. (**C**) Average ITC of Sensory ICs minus average ITC of Sensorimotor ICs. Note that colour-coding of subtraction is ten times amplified relative to panels A and B. 0 ms in plots (**A,B**, and **C**) indicate stimulus onset (**D**). Average ITC over 132 Motor ICs. (**E**) Average ITC over 343 Sensorimotor ICs. (**F)** Average ITC of Motor ICs minus average ITC of Sensorimotor ICs. Note that color-coding of subtraction is ten times amplified relative to panels D and E. 0 ms in plots (**D,E**, and **F**) indicate motor onset (button-press event). Panels C and F were masked according to two-sample t-test with p-value threshold < 0.01. The black dashed rectangles represent the same time-frequency windows of interest as in Fig. 3.

By establishing a threshold for Sensory and Motor ITC values, we separated all ICs in the study into four groups (Fig. 4A). Those ICs with Sensory and Motor ITC values exceeding the threshold (red dots in Fig. 4A; also Figs 3A and 5) were identified as Sensorimotor ICs. Those ICs with only Motor ITC value exceeding the threshold (blue dots in Fig. 4A; also Figs 3B and 5) were identified as Motor ICs. Those ICs with only Sensory ITC value exceeding the threshold (green dots in Fig. 4A; also Figs 3C and 5) were identified as Sensory ICs. ICs with neither Sensory nor Motor ITC values exceeding the threshold (black dots in Fig. 4A; also Fig. 3D) were identified as Unspecified ICs.

For classification of ICs into the four groups and further localization of Sensorimotor ICs, we had to select an ITC threshold. At sensory and motor ITC thresholds of 0.2, we observed a maximal number of sensory ICs (green lines in Fig. 4B) and a near-maximal number of motor ICs (blue lines in Fig. 4B). Therefore, we selected this value for classification of ICs into Motor, Sensory, Sensorimotor, or Unspecified. With this choice of critical threshold values, we observed on average 5.8 Sensorimotor ICs in each subject-session combination, which was in between the number of Sensory and Motor ICs (i.e. a substantial portion of the total).

### Does number of channels in EEG recordings influence number of Sensorimotor ICs?

One potential concern was that there might have been a minimum number of ICs necessary to fully resolve the brain processes^32^. Because the number of ICs produced was equal to the number of EEG channels, the number of Sensorimotor ICs observed may have been dictated by the number of EEG channels used. To address this, we examined data recorded from subjects with 64- and 127-channel EEG systems, using the same experimental design twice, and additionally extracted a subset of 32 channels from the 64-channel EEG recordings. The extraction was performed after filtering and downsampling but before the ICA procedure. The subsets of 32 channels had the same head coverage as 64-channel recordings, but with half of the electrode density, resulting in a total of twenty-one 32-channel datasets, twenty-one 64-channel datasets, and seventeen 127-channels datasets. We processed all three types datasets (32, 64, and 127 channels) applying the same data-processing procedure in order to find out whether the number of channels influences the number of Sensorimotor ICs. We applied a series of ITC thresholds from 0 to 1 with a step of 0.02 to all accepted ICs in the study (1412 ICs) and plotted the results of separation of ICs into the four groups (Motor, Sensory, Sensorimotor, or Unspecified) in Fig. 4B. Colours of the lines in Fig. 4B are congruent with colours of the dots in Fig. 4A; solid lines in Fig. 4B represent 127-ch. datasets, dashed lines represent 64-ch. datasets, and dotted lines represent 32-ch. datasets. Notably the red solid, dashed, and dotted lines in Fig. 4B are overlaying, suggesting that the number of Sensorimotor ICs does not correlate with the number of channels in an EEG system. This supports the interpretation that these are genuine Sensorimotor ICs.

### Localization of sensorimotor ICs

**Figure 6:**
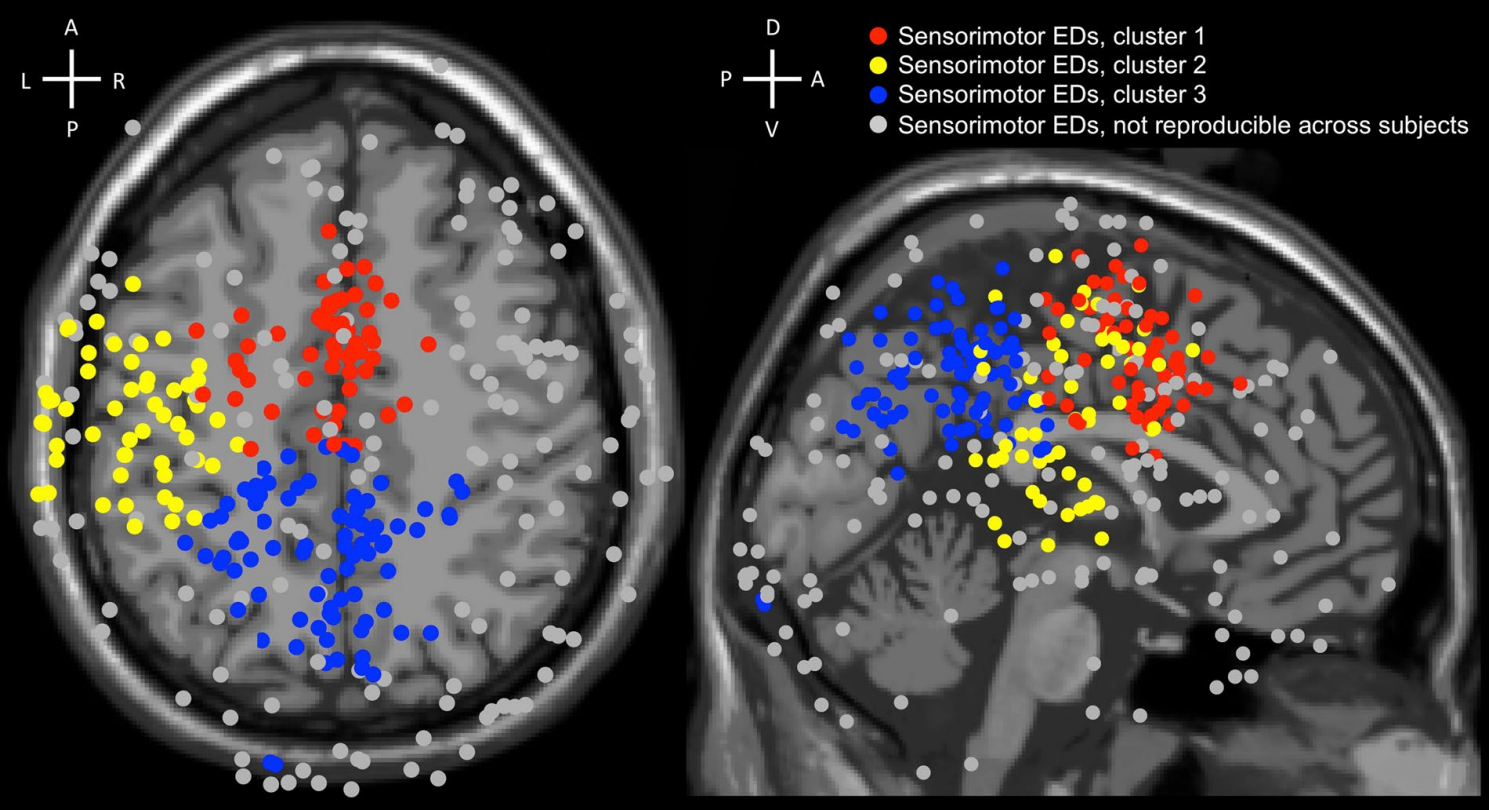
Three clusters (coloured in red, yellow, and blue) obtained by the k-means clustering algorithm from 192 Sensorimotor EDs reproducible across subjects from 32-, 64-, and 127-channel datasets. 151 Sensorimotor EDs, which were not reproducible across subjects, were coloured in grey and did not participate in the k-means clustering. Cluster 1 = Sensorimotor EDs coloured in red; Cluster 2 = Sensorimotor EDs coloured in yellow; Cluster 3 = Sensorimotor EDs coloured in blue. Labels at the crosses represent anterior (A), posterior (P), dorsal (D), ventral (V), left (L), and right (R) sides.

Where are the Sensorimotor ICs located? For each IC, we found an equivalent dipole (ED). In order to check whether locations are reproducible across subjects, we marked as reproducible only those Sensorimotor EDs which had neighbouring dipoles of at least 50% of subjects within a given radius R (coloured dots in Fig. 6). We marked Sensorimotor EDs which had neighbouring dipoles of less than 50% of subjects within a given radius R (grey dots in Fig. 6) as not reproducible across subjects. Moreover, a large part of non-reproducible Sensorimotor EDs were located outside of the cortex. To estimate a meaningful order of magnitude for the radius R, we calculated the volume of the brain accounted per Sensorimotor ED and extracted the radius of the sphere of the derived volume: V_sphere_ = V_brain_/N; where V_sphere_ = (4*π*R^3^)/3; V_brain_ = 1202 cm^3^ − average volume of a human brain across genders^34^; N = 5.8 − average number of Sensorimotor ICs in a dataset in the study (Table 1). These calculations resulted in R = 36.7 mm. The average volume of a human brain is the sum of the grey and white matters. To have a conservative selection, we took 75% of this radius (which a bit less than half of the volume), which resulted in R = 27.5 mm. Based on this criterion, 151 Sensorimotor EDs were marked as not reproducible across subjects (grey dots in Fig. 6) and were excluded from further analysis. The previous rejections of artefact-related ICs (Fig. 1) were based on individual sessions. Here we use spatial information of repeated sessions, completing the selection process of ICs. Complementary, 192 Sensorimotor EDs were marked as reproducible across subjects (coloured dots in Fig. 6) and were analysis subsequently.

**Table 1:** Average number of ICs per session ( ± s.e.m.) in 32-, 64-, and 127-channel datasets and in the four groups, given the sensory and motor ITC thresholds = 0.2.

At ITC threshold = 0.2	32 channels	64 channels	127 channels
Sensorimotor ICs	5.8 ± 0.6	5.8 ± 0.6	5.8 ± 0.8
Sensory ICs	8.2 ± 0.7	11.0 ± 0.8	11.5 ± 1.4
Motor ICs	1.9 ± 0.3	2.5 ± 0.4	2.4 ± 0.5
Unspecified ICs	4.0 ± 0.5	6.0 ± 0.7	7.5 ± 1.0
All groups, good ICs	20.0 ± 0.8	25.2 ± 1.3	27.3 ± 2.5

We used the 192 Sensorimotor EDs that were marked as reproducible across subjects in the k-means clustering. We set the number of clusters equal to 3 because the average number of Sensorimotor EDs reproducible across subjects per dataset was equal to 3.25 (192 Sensorimotor EDs divided by 59 datasets). Cluster 1 had its centroid at MNI = [−4 −2 45], Talairach = [−3 0 41] (http://sprout022.sprout.yale.edu/mni2tal/mni2tal.html)^35^, which is left cerebrum, limbic lobe, cingulate gyrus, Brodmann area 24 (www.talairach.org/applet/)^36, 37^; and encompasses 58 Sensorimotor EDs (0.98 per dataset). Cluster 2 had its centroid at MNI = [−50 −18 30], Talairach = [−48 −17 29], which is left cerebrum, frontal lobe, postcentral gyrus, white matter; and encompasses 53 Sensorimotor EDs (0.90 per dataset). Cluster 3 had its centroid at MNI = [−5 −51 38], Talairach = [−4 −48 36], which is left cerebrum, parietal lobe, precuneus, Brodmann area 31 (on the border with the posterior cingulate cortex); and encompasses 81 Sensorimotor EDs (1.37 per dataset). Two of the derived clusters were mostly symmetrical along the sagittal plane, and one cluster was located in the left hemisphere.

## Discussion

Our study revealed EEG correlates of sensorimotor processing in the human brain. Consistently across subjects, we found Sensorimotor ICs (Fig. 3A) with both event-related ITC responses tied to the stimulus onset and to the button-press event. We interpret these results as the Sensorimotor ICs represent functional neural modules that were active in both phases of sensorimotor processing. Thus, the findings support the second hypothesis that the human brain directly couples sensory recognition and motor actions rather than sequentially separating them. Our findings are compatible with alternative frameworks like embodied cognition^8^, sensorimotor contingency^19^, and common coding^13^. However, we also have found roughly as many Motor and Sensory ICs as Sensorimotor ICs. Thus, our findings do not exclude the classical view, which conceptualizes sensorimotor processing as a series of functional modules activated in a sequence from sensory input to motor execution.

The number of ICs in the Motor and Sensory groups increased with a higher number of channels, from 32 to 64 channels (Table 1). However, the increment of the number of channels from 64 to 127 did not lead to a noticeable increment of the number of ICs in these groups (Table 1). These results suggest that the number of independent sources in the brain that were involved in the sensorimotor task in our experiment and can be identified with ICA, was limited. Therefore, increasing the number of EEG channels beyond a certain number for stationary subject experiments does not lead to an increase of the number of Sensory, Motor, or Sensorimotor ICs. This is in agreement with previous work showing a functional maxima in the number of channels necessary^32^.

ICA could have potentially failed to separate sensory and motor sources. Therefore, the presence of such sensorimotor components would not be due to integrated sensory and motor processing, but to failure of ICA to separate these two separate processes. Even then, a joint IC would suggest the colocalization of such processes, because ICA components are spatial filters. To address this potential issue, we recorded subjects with two EEG systems with different number of electrodes, and additionally we analysed a subset of 32 channels from 64-channels datasets. The number of Sensorimotor ICs did not correlate with the number of channels in an EEG system, supporting our expectation that these are genuine Sensorimotor ICs.

The inverse problem of source localization and possible artefacts within ICA, as well as our assumption that ICs can be represented by a single equivalent-dipole model, are potential limitations which constrain interpretation of the current results. We also do not know the precise properties of such ICs within the frameworks of embodied cognition^8^, sensorimotor contingency^19^, and common coding^13^. If sensorimotor functional modules (represented by Sensorimotor ICs in the study) form a complex and distributed structure then the generation of more complex higher-order electric fields, e.g., a quadrupole, is possible. A dipole fit to such ICs would have a high residual variance and lead to a rejection by the dipole fit filtering procedure. However, in the current study we report the existence of sensorimotor functional modules represented by Sensorimotor ICs; thus, we deliberately chose the conservative approach to minimize the chances of false positive results and be sure that such Sensorimotor ICs are real.

The topographical sparseness threshold used to remove ICs from further analysis was set to 5 (Fig. 2). We did an additional control to check whether the threshold TS = 5 might cause a selection bias on components selected for further analysis. Those results indicated that the threshold choice did not cause a selection bias. We found that TS threshold selection below 5 did not influence the proportion of Sensorimotor ICs (Sensory and Motor ITC values > 0.2) in the 32-, 64-, and 127-channel datasets. Having a TS threshold higher than 5 was not appropriate because we wanted to remove from further data analysis ICs which had a one-channel-dominant-projection-weight topography. In 32-channel datasets, such a topography corresponds to TS = 5.5. Therefore, the current selection TS = 5 seems reasonable and did not cause a selection bias on Sensorimotor ICs selected for further analysis.

The ITC threshold used to divide all ICs into the four groups (Sensorimotor, Sensory, Motor, and Unspecified - Figs 3 and 4) was set to 0.2. Figure 4 shows how the distribution of ICs into Sensory, Motor, and Sensorimotor groups changes when the ITC threshold moves. There was no gap of bi-modal distribution, and we did not find indications that the results depended on the exact ITC threshold. The selected ITC threshold = 0.2 seems to be a reasonable choice, as it is around the peaks of Sensory (green) and Motor (blue) lines (Fig. 4B). Furthermore, also for deviant choices of the threshold value, the number of Sensorimotor ICs was minimally dependent on the number of channels (Fig. 4B, solid, dashed, and dotted red line). Thus, we did not find any indication that Sensorimotor ICs were a result of squeezing sensory and motor modules into a single IC due to a limited number of channels. Instead, additional degrees of freedom were taken by the Sensory ICs and the Unspecified ICs. The ITC threshold level = 0.2 gave a good separation of ICs into the groups and shifting the threshold slightly up or down did not change the ratio of Sensorimotor ICs in datasets with a different number of channels (32, 64 and 127 ch.).

Location of the centroid of Cluster 1 coincides with the anterior cingulate cortex (ACC). The anterior cingulate cortex is associated with conflict monitoring in the engagement of cognitive control^38, 39^, i.e., in the Stroop task^40^. Indeed, the paradigms in the current study are variations of the Stroop task. It has also been proposed that “the anterior cingulate cortex is a cortical region where a cognitive/motor command, coming from a different cortical region (e.g., prefrontal cortex), is being modulated and funnelled to the motor system, and this modulation takes place within distinct, motor output-specific subregions of the anterior cingulate cortex, thus emphasizing the motor character of this region”^41–43^. Errors in the localization accuracy of EDs are possible, limiting our conclusions to the anterior cingulate cortex likely being involved with sensorimotor processing for our task.

The centroid of Cluster 2 lies in white matter in the vicinity (±10 mm) of Brodmann areas 1, 2, 3, 4, 6, and 40^35^. Activity of the primary motor and somatosensory cortices could explain motor event-related ITC response of Sensorimotor ICs in Cluster 2. Indeed, the index and the ring fingers of the right hand were used in the experiment to conduct motor responses. However, Sensorimotor ICs have sensory components as well, and the stimuli were centrally presented, which does not correlate with the leftward bias. Thus, it is not completely obvious why using the right hand should lead to a bias towards left hemispheric Sensorimotor ICs.

Location of the centroid of Cluster 3 coincides with the precuneus, in the area bordering the posterior cingulate cortex. Both the precuneus and the posterior cingulate cortex are associated with high-level behavioural correlates, as well as being a part of an attentional system^44, 45^. An fMRI study of Stroop tasks proposed that the precuneus is a part of a system which is responsible for executive aspects of attentional selection, in particular, sets a top-down bias for selecting certain types of information, e.g., colour^46^. Another event-related fMRI study where subjects performed a variation of the Wisconsin Card Sorting Test concluded that an increase in neural activity in the precuneus correlates closely with the execution of attentional shifts between object features^47^. The complex speeded-response tasks in the current study require attentional resources to compare and match stimuli features, as well as a top-down bias for selecting relevant information. Therefore, event-related ITC responses of Sensorimotor EDs from Cluster 3 during sensory, as well as motor processing, allow the assumption that the attentional system is involved in sensorimotor processing.

**Figure 7:**
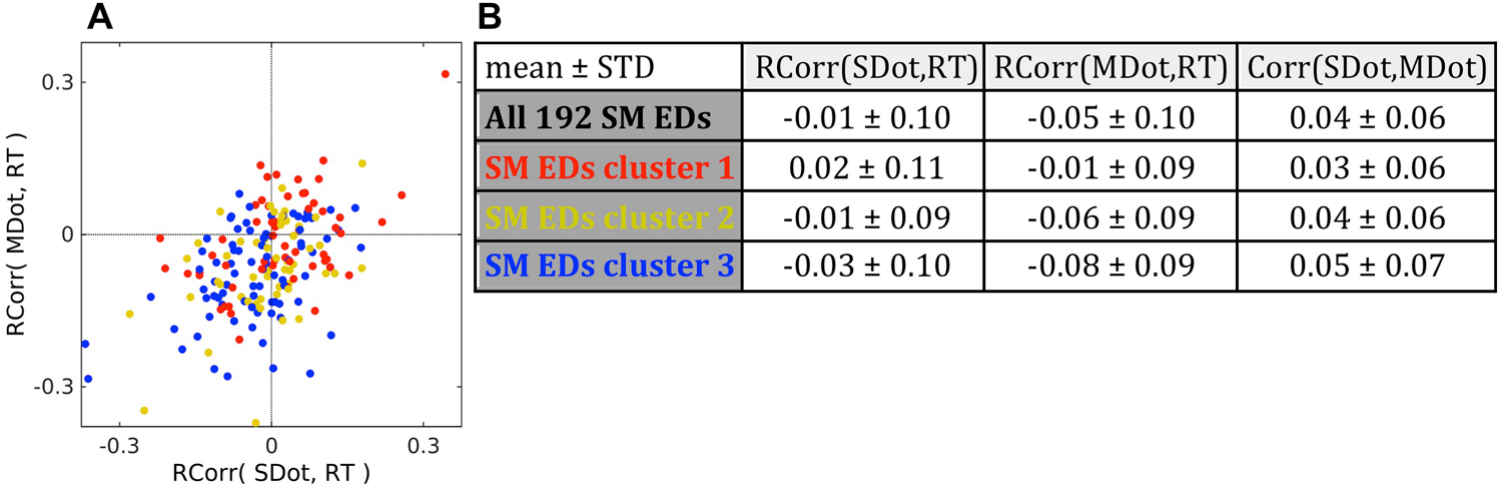
Rank correlation of sensory and motor related amplitudes in single trials with reaction time. **(A)** Scatter plot with 192 points representing all Sensorimotor (SM) EDs. Red points represent Sensorimotor EDs in cluster 1 (58 SM EDs). Yellow points represent Sensorimotor EDs in cluster 2 (53 SM EDs). Blue points represent Sensorimotor EDs in cluster 3 (81 SM EDs). The OX axis represents rank correlation (Spearman’s rho) of reaction times (RT) in trials and dot products (SDot) of the sensory related ERP and single trials on the interval from 100 ms to 300 ms after the stimulus onset in a SM IC. The OY axis represents rank correlation of reaction times in trials and dot products (MDot) of the motor related ERP and single trials on the interval from −100 ms to 100 ms around the motor onset in a SM IC. **(B)** Table of mean values ± STD of correlation coefficients for all 192 SM EDs and for individual clusters depicted in Fig. 7A. RCorr(SDot,RT) represents rank correlation (Spearman’s rho) of sensory related dot products in trials and reaction time in trials. RCorr(MDot,RT) represents rank correlation of motor related dot products in trials and reaction time in trials. Corr(SDot,MDot) represents linear correlation of sensory and motor related dot products in trials. Negative rank correlation coefficients reflect a correspondence of higher amplitude (dot product) with shorter reaction time in trials in an IC. We calculated the mean values without application of Fisher z-transformation, as the extreme values of correlation coefficients were between −0.4 to 0.4 with the highest density around zero.

Rank correlation (Spearman’s rho) showed that some Sensorimotor ICs have a noticeable correlation between reaction time and the amplitude of sensory or motor response in a trial. However, Sensorimotor ICs did not show any prominent shift towards the positive or negative direction. Mean values of all Sensorimotor EDs as well as their sub clusters 1, 2, and 3 lie within one standard deviation from zero. Cluster 3 had the highest negative rank correlation coefficients −0.08 ± 0.09 STD (Fig. 7) for motor responses. Negative rank correlation coefficients in Fig. 7 indicated a correspondence of higher amplitude (dot product) with shorter reaction time in trials in an IC. Overall, there was a positive correlation of sensory and motor dot products in ICs, which means that rank correlation sign had a higher probability to be the same for sensory and motor rank correlations with reaction time in an IC. It has been shown before that some ICs may have both sensory and motor component^48^. However, as far as we know it has not been systematically studied as a primary variable.

There are other noninvasive imaging techniques that can quantify brain activity, like functional magnetic resonance imaging (fMRI)^49^. An fMRI study would give a higher spatial resolution and more precise localization and structure of sensorimotor functional modules in the brain. However, for the analyses highlighted here, resolution in the temporal domain is a critical feature. In BOLD fMRI, the response is a smooth continuous function and a faster sampling than once per second yields only linear interpolation, since a hemodynamic response lasts over 10 seconds^49^. Thus, given that the average reaction time in the study was 515 ms, it would be difficult or impossible to differentiate sensorimotor processes from sensory processes and motor processes by means of fMRI measurements in the current setup. Recordings from an electrode array in the brain tissue may provide both, good spatial and good temporal resolution, but they require either animal or clinical studies. Finding the exact structure and localization of sensorimotor functional modules are important questions for future research.

Several fMRI studies reported increased activity of the anterior cingulate cortex, the posterior cingulate cortex, and the precuneus during the execution of Stroop tasks^42, 43, 46, 50^. These areas are compatible with a rich spectrum of functional significance, including attention, self-consciousness, conflict monitoring, and cognitive control^2^. These findings support our results, since these brain areas coincide with Cluster 1 and Cluster 3 centroids.

Different types of sensorimotor tasks (i.e., whole body movement) also activate brain areas which coincide with the detected clusters of Sensorimotor ICs in the current study. A study of vestibular sensory processing with motor tasks revealed that impending loss of balance is associated with electrocortical dynamics in the anterior cingulate, posterior cingulate, anterior parietal, bilateral sensorimotor, and superior dorsolateral-prefrontal cortex^51^. Interestingly, that study also found a left hemisphere bias in sensorimotor IC clusters. Another EEG study showed that the premotor and parietal areas are involved in visually guided gait in humans. Mu, beta, and lower gamma rhythms in premotor and parietal cortices are suppressed during conditions that require an adaptation of steps in response to visual input. The study concluded that “activity in the parietal cortex likely reflects direct visuomotor transformations required by the task. Increased activity in the premotor cortex may indicate motor planning involved in adapting the steps to the visual input”^52^. Thus, different types of tasks, as well as processing in different sensory modalities coupled with motor tasks, correlate with electrocortical dynamics in the brain areas related to the detected clusters of Sensorimotor ICs in the current study.

The present results are congruent with multiple frameworks emphasizing a coupling of sensory and motor brain processes. The embodied cognition framework, espousing that perception guides motor actions and motor actions influence perception is one such conceptual theory. Another is the sensorimotor-contingency theory^19^, which emphasizes the key role of action for perception. Our results show bias in Sensorimotor ED locations toward the left hemisphere (Fig. 6). One reason for that might be that the right hand was used in the experiment to conduct motor responses. However, Sensorimotor ICs have sensory components as well, and the stimuli were centrally presented, which does not correlate with the leftward bias. This is congruent with the “stimulus-response compatibility” effect which is explained by the common-coding theory put forward by Wolfgang Prinz^13, 14^. This theory is built on the idea that sensory and motor codes share some common features and that perceptual and motor representations are linked. Thus, according to the common-coding theory, there are some neural substrates involved in the decoding of sensory information coding of motor actions. Sensorimotor ICs in the current study have strong event-related responses after a stimulus onset as well as at the moment of a button-press event. However, additional processing at higher cortical areas may be required to clarify decoded information and modulate to an appropriate action. This may lead to a certain variance in reaction time and a delay between sensory and motor event-related responses of Sensorimotor ICs. Other evidence for this common-coding theory comes from observations in human and rhesus monkeys where the same neurons responded after a stimulus onset and timed to the motor response^53–55^. Combined with the current results, it is possible to interpret the findings as supporting functional modules directly coupling sensory and motor processes.

In this study, we consistently found across subjects a substantial number of Sensorimotor ICs, in which event-related ITC responses are related to both sensory stimulation and motor response onsets. Sensorimotor ICs are possible EEG correlates of sensorimotor processing in the human brain. We hypothesised that these Sensorimotor ICs represent functional modules in the brain, combining sensory recognition and motor responses. This is compatible with such frameworks as embodied cognition^8^, sensorimotor contingency^19^, and common coding^13, 14^. Future studies could expand understanding of functional meaning of sensory and motor processing in such Sensorimotor ICs using different tasks and trying to more precisely identify their neuronal sources.

## Methods

### Participants and EEG recordings

Twenty-one paid healthy volunteers (eleven males), aged 19–29 years (mean = 24, sd = 3.1), participated in the EEG study. All participants had normal (self-reported) or corrected-to-normal vision. We obtained written informed consent from all subjects prior to the experiment and the protocol had been approved by the review board of the University of Osnabrück. Experimental procedures conformed to the Declaration of Helsinski and national guidelines. This project was a part of a larger study, in which participants were recorded two times in the same EEG experiment but with different EEG system. In the following, it was important for us that these EEG systems had a different number of channels. The first session was recorded with a 64-channel ActiCAP EEG system and the second session was recorded with a 127-channel ASA-Lab EEG system on a different day. These two EEG systems provide statistically indistinguishable EEG data^56^. Seventeen participants were recorded in both sessions and four participants were recorded only in the first session.

### Stimuli and tasks

**Figure 8:**
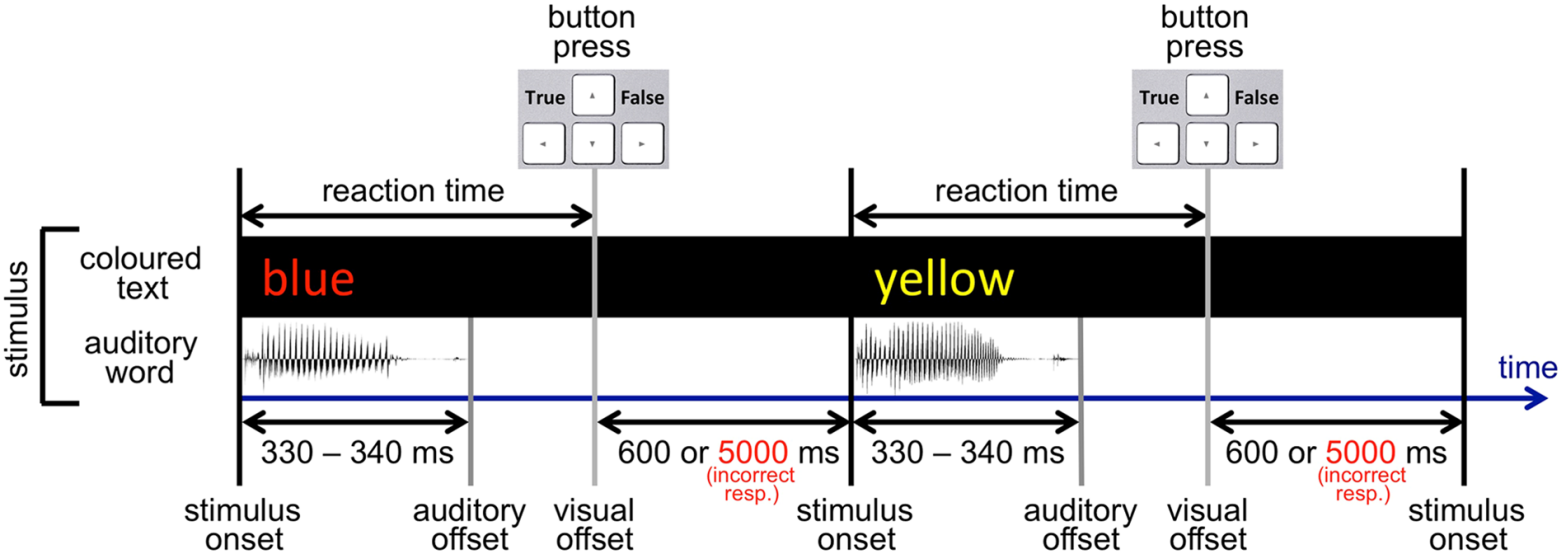
Experimental setup. A stimulus is a coloured text appearing on a monitor simultaneously with an auditory word through a headset. In a reaction-time task, subjects had to categorize each stimulus by pressing Arrow Left (True) or Arrow Right (False) on a keyboard according to a given categorization rule in each block. Please note, that the figure represents the first half of trials of each block, in the second half of trials of each block, the disappearance of the visual part of the stimulus (visual offset) occurred after a short delay of 150 ms after a button-press event (in the case of a correct response).

The stimuli were a combination of a coloured text (visual angle: 2 × 1°) on a black background presented centrally on a computer screen and a simultaneously occurring auditory word through a headset (Fig. 8). Thus, each stimulus contains three sources of information about a colour, which may or may not match. The experiment was conducted in German; four different colours were used: rot (German red), grün (German green), blau (German blue), and gelb (German yellow), yielding 64 possible combinations. The setup is reminiscent of the Stroop effect paradigm^40^, but is a distant variation of it due to containing an auditory component as well as different tasks and type of responses.

Each recording consisted of 5940 trials (one stimulus and one response per trial) divided into 6 blocks with breaks in between. One categorization rule per block was used. The categorization rule in Block 1: to press Arrow Left (True) when colour = red and text = red, Arrow Right (False) in other cases; in Block 2: to press Arrow Left (True) when colour = red and sound = red, Arrow Right (False) in other cases; in Block 3: to press Arrow Left (True) when text = red and sound = red, Arrow Right (False) in other cases; in Block 4: to press Arrow Left (True) when colour = text, Arrow Right (False) in other cases; in Block 5: to press Arrow Left (True) when colour = sound, Arrow Right (False) in other cases; in Block 6: to press Arrow Left (True) when text = sound, Arrow Right (False) in other cases. Participants categorized each stimulus in accordance with a given rule by pressing on a keyboard Arrow Left with the index finger of the right hand or Arrow Right with the ring finger of the right hand. 50% of trials in each block belonged to one of the two categories. The two categories appeared in a random sequence. We pooled all six blocks to get more data for the ICA and ITC analyses.

The duration of the auditory part of a stimulus were as follows: rot (German red) = 340 ms, grün (German green) = 340 ms, blau (German blue) = 340 ms, gelb (German yellow) = 330 ms. The visual part of the stimulus were presented on the monitor until a button-press event occurred. We measured reaction time from the common onset of the auditory and the visual parts of a stimulus and until a button-press event. In the case of a correct response, the coloured word disappeared from the screen for 600 ms, and after that, a new stimulus appeared. In the first half of trials of each block, the disappearance of the visual part of the stimulus occurred immediately after a button-press event (in the case of a correct response). In the second half of trials of each block, the disappearance of the visual part of the stimulus occurred after a short delay of 150 ms after a button-press event (in the case of a correct response). In the case of an incorrect response, the coloured word stayed on the screen unchanged for 5000 ms to provide feedback to the participant.

Participants were motivated to give a correct response as quickly as possible. They always had the possibility to press a pause button and check their current mean reaction time and error-response statistics, as well as to compare their reaction time to that of other participants, which increased their motivation to give a correct response as quickly as possible. Subjects could also earn a bonus of up to 30% of the basic payment; the shorter the reaction time, the bigger the payment. However, in order to ensure accuracy, if the number of incorrect responses exceeded 5%, they lost their bonus completely, as well as being moved down in the reaction-time score table. Therefore, the usual strategy was to shorten reaction time down to an efficient level, where the number of incorrect responses did not exceed 5%. The mean number of error responses per session was 4.1% ± 1% (STD). All participants got a short practice before the real experiment to learn the strategy and reach a plateau of performance.

The following trials were discarded from EEG-data processing and reaction time analysis: each ten first trials after the beginning of each new block; trials with error responses, as well as two trials after them; one trial before and three trials after each pause (subjects were able to pause the experiment anytime). The mean number of discarded trials per recording was 14.7% ± 3.5% (SD). All not discarded trials were marked as valid.

**Figure 9:**
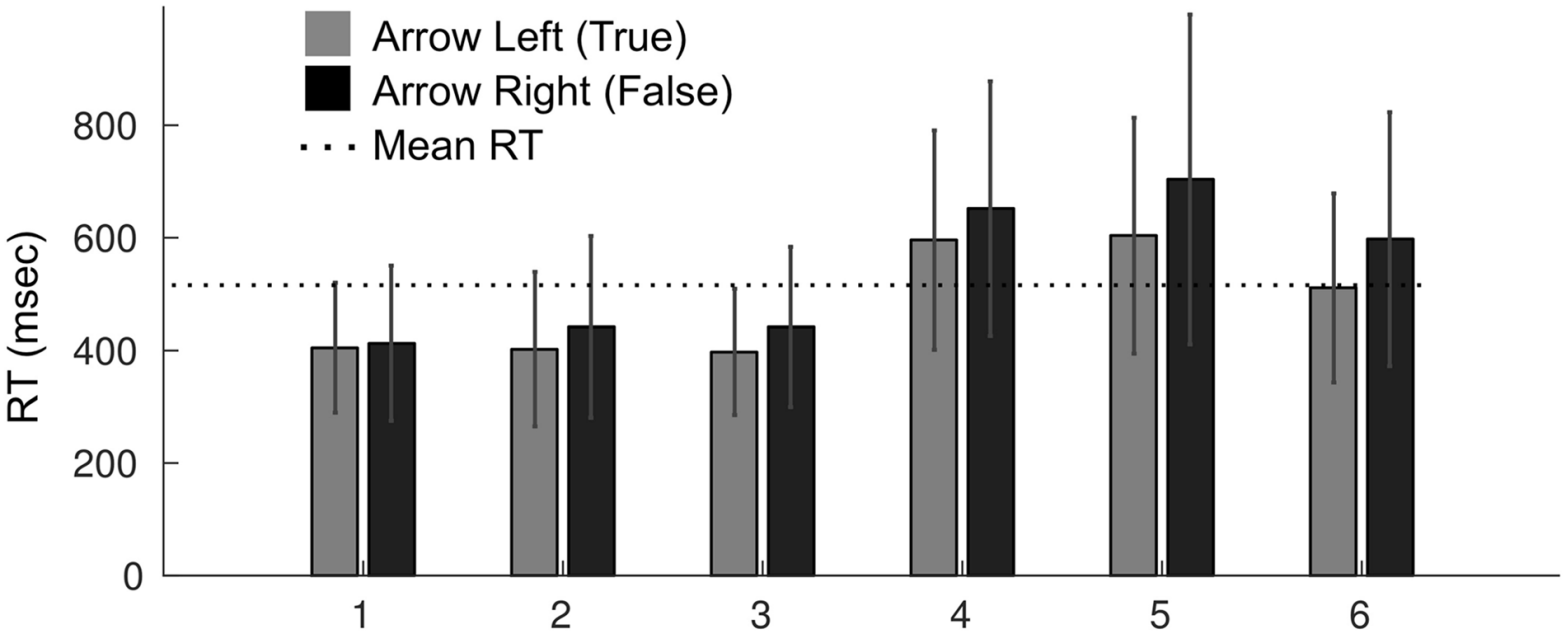
Reaction time for button presses Arrow Left (True) and Arrow Right (False) in the six blocks. The dotted line depicts the grand mean RT (515 ms) in the study. The X-axis depicts block ID. The Y-axis depicts the mean reaction time (RT) of all respective trials across all recording sessions. Error bars represent standard deviation.

We measured reaction time as the time from the common onset of the auditory and the visual stimulus components until a button-press event occurred (Fig. 8). The grand mean reaction time of valid trials (not discarded) across all thirty-eight recording sessions was 515 ms ± 213 ms (STD) (dotted line in Fig. 9). The mean reaction time for “Arrow Left (True)” categorizations was 486 ms ± 184 ms (STD) and for “Arrow Right (False)” categorizations was 543 ms ± 236 ms (STD). The mean reaction time for “Arrow Left (True)” and for “Arrow Right (False)” categorizations in the six blocks was the following (Fig. 9): in Block 1, 405 ms ± 115 ms (STD) and 413 ms ± 138 ms (STD) respectively; in Block 2, 402 ms ± 137 ms (STD) and 442 ms ± 161 ms (STD) respectively; in Block 3, 397 ms ± 112 ms (STD) and 442 ms ± 142 ms (STD) respectively; in Block 4, 596 ms ± 195 ms (STD) and 652 ms ± 226 ms (STD) respectively; in Block 5, 604 ms ± 209 ms (STD) and 704 ± 293 ms (STD) respectively; in Block 6, 511 ms ± 168 ms (STD) and 598 ms ± 225 ms (STD) respectively.

### EEG-data processing

EEG-data processing and analysis was performed using EEGLAB^26^ in Matlab. Each recording was filtered with a 3–45 Hz bandpass finite impulse response filter. Sampling rate was reduced from 1000 to 500 Hz for 64-channel ActiCAP EEG system and from 1024 to 512 Hz for 127-channel ASA-Lab EEG system. Noisy periods of EEG data were manually marked and cut out. The EEG data after elimination were processed in each recording by ICA using EEGLAB’s ‘runica’ function^24–26^. Further data analysis and results were based on the resulting ICs and their activity. Please note, “as scale and polarity information is distributed in the ICA decomposition (not lost!) between the projection weights (column of the inverse weight matrix, EEG.icawinv) and rows of the component activations matrix (EEG.icaact), the absolute amplitude and polarity of component activations are meaningless and the activations have no unit of measure (through they are proportional to microvolt)” (https://sccn.ucsd.edu/wiki/Chapter_10:_Working_with_ICA_components).

### Calculation of Sensory and Motor ITC values of ICs

We calculated the ITC of all trials in a session two times per IC (Fig. 3): the first time, for stimulus alignment of trials and the second time, for button-press alignment of trials. ITC calculation was done in EEGLAB using the function ‘pop_newtimef’ with two-tailed permutation significance probability test (‘alpha’ = 0.01). Non-significant features of the output were zeroed out and plotted in green. We measured the maximum ITC value in relevant time-frequency windows of interest. For stimulus-onset-aligned trials, the time-frequency window of interest was from 100 ms to 300 ms and from 3 Hz to 15 Hz. The maximum value of ITC in the time-frequency window of interest was assigned as the Sensory ITC value of the IC (Fig. 3). For motor-response-onset-aligned trials, the time-frequency window of interest was from −100 ms to 100 ms and from 3 Hz to 15 Hz. The maximum value of ITC in the time-frequency window of interest was assigned as the Motor ITC value of the IC (Fig. 3). Thus, each IC had sensory-related and motor-related ITC values, which served as scalar measures of sensory and motor event-related responses of an IC, respectively. This kind of dual-analysis of stimulus-locked and response-locked trials is also common in monkey neurophysiology saccade experiments with intracranial recordings in order to differentiate neurons that are sensory- or motor-potential related^3^.

### Equivalent dipoles localization

In order to investigate localization of ICs, an equivalent dipole (ED) was calculated for each IC using the ‘Fine fit’ procedure for a single dipole model in the “DIPFIT v2.3” toolbox (EEGLAB). The head model ‘standard_vol.mat’ and the MRI file ‘standard_mri.mat’ (MNI Colin27)^57^ provided in the toolbox were used as the basis. Since we do not have MRI data from subjects, we chose this relatively simple method of dipole localization. Another option would be to calculate dipole density^58^ using dipole density function of EEGLAB. Prior to each EEG recording we digitized the 3D positions of all electrodes and three major fiducials (nasion, left and right preauricular points) using the optical ANT Neuro xensor^TM^ system (ANT Neuro, Enschede, Netherlands; www.ant-neuro.com/products/xensor). We warped these positions of electrodes to fit the MNI Colin27 template afterwards. Five reference points were used to obtain the best fitting of a recorded electrode positioning model and the head model: three fiducial points (nasion, left and right preauricular), a position of the most posterior electrode, and a position of the uppermost electrode. After the best fitting between the head model and a recorded electrode positioning model was achieved (by moving, rotating, and resizing of the electrodes model along axes), electrodes were projected onto the head model. If residual variance of an ED exceeded 15%^29–31^, then the corresponding IC was marked as residual-variance-bad and excluded from further data analysis (Fig. 2).
